# Initial Assessment of Alcohol-Related Psychiatric Admissions: A Clinical Audit From Malta

**DOI:** 10.1192/bjo.2026.11720

**Published:** 2026-06-30

**Authors:** Elisa Farrugia Pace, Sean Caruana Scicluna, Emma Camilleri

**Affiliations:** 1 Mater Dei Hospital, Msida, Malta; 2 Mount Carmel Hospital, Attard, Malta

## Abstract

**Aims:**

Chronic alcohol dependence increases the risk of alcohol withdrawal syndrome, requiring prompt identification. Despite this, no locally endorsed clinical guidelines currently exist to support clinicians. This audit aims to evaluate the adequacy of alcohol screening and initial clinical assessment at admission to an acute psychiatric inpatient service, focusing on the identification and management of AUD, alcohol dependence, and alcohol withdrawal, and compares current practice with international standards.

**Methods:**

A retrospective audit of all acute psychiatric admissions in March 2025 was conducted. Data were extracted in line with the *American Society of Addiction Medicine Clinical Practice Guideline for Alcohol Withdrawal Management*, including documentation of alcohol use, clinical history, withdrawal features, and relevant investigations. All data were anonymized following ethical approval.

**Results:**

Of the 217 total admissions, 190 patients were included in the final analysis. Among the analyzed cohort, alcohol use was documented in 132 patients (70%), with 36 individuals (27.3%) confirming regular consumption.

Notably, medical and surgical comorbidities were recorded in 100% of cases (n=36), followed by age at 95.6% (n=35), and suicide risk assessments at 91.7% (n=33). Clinicians also frequently screened for other substance use (100%) and history of mental illness (88.9%). Regarding consumption patterns, while the quantity of alcohol was frequently noted (88.9%), details regarding the duration of dependency and the specific time of last consumption were documented in only 8.3% of files.

Despite the use of CIWA-A scoring in 66.7% of regular users, documentation for previous withdrawal delirium, history of withdrawal seizures, and observed autonomic activation during the initial interview all stood at 0%.

Furthermore, urine toxicology was performed for 63.9% of patients. Metabolic and infectious screening was inconsistently and were documented as following; LFTs 52.8% (n=19), Albumen 41.67% (n=15), INR 27.8% (n=10), Ethanol 36.11% (n=13), Pregnancy test 10% (n=1 out of females in the cohort) and HIV and Hepatitis Screen within 5 years 55.56% (n=20).

**Conclusion:**

In this retrospective audit, significant shortcomings were identified. These findings indicate incomplete adherence to internationally recommended assessment standards and may limit the early identification of alcohol withdrawal risk. Implementation of a locally adapted alcohol assessment guideline consistent with international best practice is therefore warranted. Re-audit is recommended to assess changes in clinical practice and to evaluate associations with patient-related outcomes. No financial support was received for this study.

